# Aromatase Activity and Bone Loss in Men

**DOI:** 10.4061/2011/230671

**Published:** 2011-06-24

**Authors:** Daniela Merlotti, Luigi Gennari, Konstantinos Stolakis, Ranuccio Nuti

**Affiliations:** Department of Internal Medicine, Endocrine-Metabolic Sciences and Biochemistry, Viale Bracci 1, 53100 Siena, Italy

## Abstract

Aromatase is a specific component of the cytochrome P450 enzyme system responsible for the transformation of androgen precursors into estrogens. This enzyme is encoded by the *CYP19A1* gene located at chromosome 15q21.2, that is, expressed in ovary and testis, but also in many extraglandular sites such as the placenta, brain, adipose tissue, and bone. The activity of aromatase regulates the concentrations of estrogens with endocrine, paracrine, and autocrine effects on target issues including bone. Importantly, extraglandular aromatization of circulating androgen precursors is the major source of estrogen in men. Clinical and experimental evidences clearly indicate that aromatase activity and estrogen production are necessary for longitudinal bone growth, the attainment of peak bone mass, pubertal growth spurt, epiphyseal closure, and normal bone remodeling in young individuals. Moreover, with aging, individual differences in aromatase activity may significantly affect bone loss and fracture risk in men.

## 1. Background

**Figure 1:**
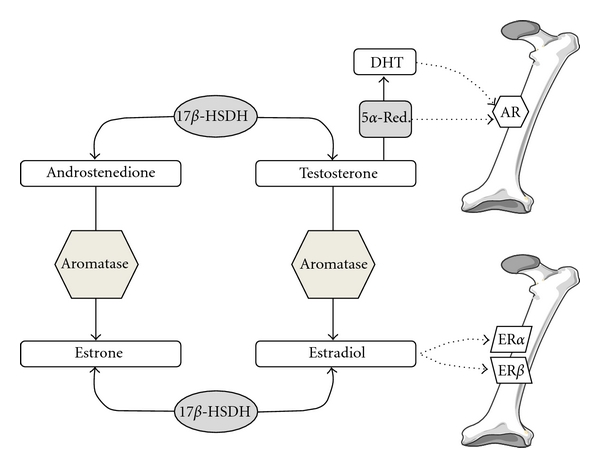
Proposed models of sex steroid hormones action on bone.

Sex steroid hormones are important for the acquisition and maintenance of bone mass in both sexes [1, 2]. Alterations in their levels can be relevant in the pathogenesis of osteoporosis and fractures because their deficiency may lead to suboptimal acquisition of peak bone mass in young individuals or bone loss in adulthood. While estrogens effects in bone in females have been well established (as estrogen deficiency after menopause leads to an imbalance between bone resorption by osteoclasts and bone formation by osteoblasts), the role for estrogen in male skeletal health has only recently become appreciated [3–6]. In fact, even though alterations in circulating androgen have been associated with low bone mass and impaired bone strength [7], their primacy has been increasingly questioned as direct and indirect evidence has emerged suggesting that estrogens also play a major role in male skeletal health [3–7]. These new observations underscore the normal biosynthetic pathway by which estrogens are made in men, via the activity of aromatase (a cytochrome P450 product of the *CYP19A1* gene) on circulating androgenic precursors (Figure 1). 

Of interest, several clinical and experimental studies on estrogen and/or aromatase deficiency also reinforced the hypothesis of a threshold estradiol level for skeletal sufficiency in the male [1–4]. In fact, either in cross-sectional or longitudinal analysis in aging men bone mineral density (BMD) and rates of bone loss at different skeletal sites were unrelated to serum estradiol concentrations when the latter were above the median value, while they were clearly associated with estradiol at concentrations below the median [8–10]. This hypothesis gained further support from a study in which raloxifene (a selective estrogen receptor modulator) was given to men of varying estradiol levels [11]. Subjects with serum total estradiol levels below 96 pmol/liter responded to raloxifene with a decrease in bone resorption markers while above this estrogen value, raloxifene caused an increase in bone resorption markers [11]. Overall, these observations clearly indicate that men need a sufficient concentration of estrogen, defined as a threshold value, for normal skeletal remodeling. In all these studies, the required concentration appeared to be remarkably similar, ranging from 90 to 110 pmol/liter in case of total estradiol or from 40 to 55 pmol/liter in case of bioavailable estradiol [5]. This apparent threshold value is higher than typical estradiol concentrations for postmenopausal women who are not receiving exogenous estrogens. On the other hand, premenopausal women and young men are typically above this apparent threshold level, while up to 50% of middle-aged men fall below this estradiol threshold and, thus, are at higher risk of bone loss as shown in several cross-sectional and longitudinal studies [1–4]. Importantly, estradiol levels above a given threshold are also important to prevent fractures, as recently demonstrated in a prospective study of men from the MrOS cohort [12].

This paper summarizes the evidence that aromatase activity plays an important role in the skeleton in men, either in young individuals or in the elderly, by its actions to convert androgens to estrogens in bone and other peripheral tissues.

## 2. Aromatase and Estrogen Production in Men

Aromatase is a specific component of the cytochrome P450 enzyme system that converts the delta 4-3-one A ring of C19 androgen precursors into the corresponding phenolic A ring characteristic of C18 estrogenic compounds 

While in fertile women the ovary represents the major source of circulating estrogen, which functions as a circulating hormone to act on distal target tissues, in men the testes account at most for 15% of circulating estrogens, while the remaining 85% is due to peripheral aromatization of circulating androgen precursors in different tissues [13, 14]. These include the adipose tissue, the brain, the skin, the endothelium, and the bone. It has been demonstrated that testicular androgen precursors contribute more to the total amount of circulating estradiol than adrenal androgens [15]. In fact, dexamethasone-induced suppression of adrenal steroid synthesis moderately decreases estradiol concentrations [16], whereas orchidectomy (ORX) leads to a more dramatic suppression of plasma estradiol [17, 18]. Clearly, these extragonadal sites of estrogen biosynthesis lack the ability to synthesize C19 precursors from cholesterol, hence, their estrogen-producing activity totally depends on the availability of these circulating C19 androgenic steroids [19]. Moreover, the estrogen synthesized within these extragonadal compartments may be also locally active in a paracrine or intracrine fashion [13, 20]. 

 In human bone, aromatase is expressed in osteoblast or osteoblast-like cells from fetal and normal tissue [21–23], in articular cartilage chondrocytes, in adipocytes adjacent to bone trabeculae, and in osteocytes, but not in osteoclasts [23]. Importantly, a recent study demonstrated that aromatase gene can be expressed in bone tissue in consistent amounts, at levels similar to those found in adipose tissue [24]. In particular, osteoblasts are the major source of aromatase within the bone microenvironment [24].

**Figure 2:**
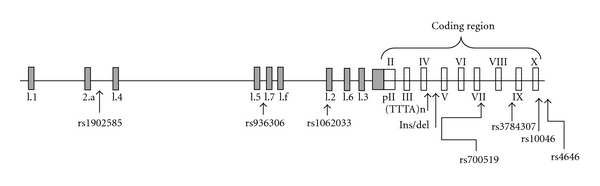
Aromatase gene (*CYP19A1*) with its promoters and untranslated first exons. Major polymorphic variants of the *CYP19A1* gene are indicated.

Aromatase is encoded by the *CYP19A1* gene located at chromosome 15q21.2 [25] (Figure 2). Despite the presence of a common *CYP19A1* gene, in a tissue-specific fashion, a number of untranslated initial exons are found in aromatase transcripts due to differential splicing by at least 10 different tissue specific promoters [26–28]. Only the 30-kb 3′ region of the gene (containing exons 2 to 10) encodes aromatase while a larger 93 Kb 5′ flanking region serves as the regulatory unit of the gene [27, 28]. Thus all the multiple exons 1 are not translated, so that the different splicing patterns lead to transcripts that are all translated as the same protein. Importantly, this complex structure of the promoter region of the gene defines the tissue-specific regulation of aromatase activity and estrogen biosynthesis. Thus, the ovary, testes, adipose tissue, brain, and bone each utilize their own promoters and associated enhancers and suppressors leading to different amounts of mRNA transcripts, mRNA stability and/or protein translation [26, 29, 30]. 

In the skeleton, the majority of aromatase transcripts contain exon 1.4 and exon 1.6 [31–33]. Some minor transcripts by promoter I.3, PII and If have been also described [31]. Interestingly, experiments in osteoblast cell lines demonstrated that cortisol may induce aromatase gene expression transiently and that 1,25 dihydroxyvitamin D can maintain its expression, dependent on vitamin D receptor density [34–36]. Moreover, a more recent study demonstrated that Runx2 (a key regulator of osteoblast differentiation) directly increases aromatase gene expression in human osteoblast cells by increasing promoter I.4 and I.6 activity [37]. In keeping with this in vitro evidence, a marked decrease in skeletal aromatase expression has been described in Runx2-deficient mice [37].

## 3. Aromatase Deficiency and the Male Skeleton

During the past 2 decades, several clinical and experimental observations underscored the importance of local and peripheral aromatization of androgens into estrogen for skeletal homeostasis in males.

### 3.1. Aromatase Deficiency Syndrome

The discovery of human cases of aromatase deficiency preceded the construction of aromatase knockout animals and, in fact, provided the first insight into the role of estrogens and aromatase in male skeletal physiology. 

Human aromatase deficiency is a very rare autosomal recessive syndrome characterized by congenital estrogen deprivation caused by loss-of-function mutations in the *CYP19A1* gene [38]. In both genders, the overall severity of the phenotype may be variable according to residual enzyme activity [38, 39]. While affected females generally have ambiguous genitalia at birth and fail to develop secondary sexual characteristics, the affected male individuals have normal male sexual differentiation and pubertal maturation, and their clinical phenotype mostly develops after puberty [5, 40]. So far, there are at least 9 known cases of inactivating mutations in the *CYP19A1* gene and aromatase deficiency in men [5, 41–49]. All these patients generally showed markedly low or undetectable estrogen levels while androgens were normal or even elevated. Interestingly, skeletal maturation and bone metabolism were severely impaired in all these subjects, with a similar phenotype to a previously described male case of loss of function mutation at the estrogen receptor alpha (*ESR1*) gene [50]. Common skeletal characteristics include tall stature and continued longitudinal growth due to unfused epiphyses, delayed bone age, lack of pubertal growth spurt, eunuchoid skeletal proportions, genu valgum, elevated bone resorption markers, and low bone mass. Moreover, other extraskeletal characteristics, such as lipid abnormalities, increased body weight, hyperinsulinemia, and various degrees of glucose impairment (including diabetes and acanthosis nigricans in 1 and 2 cases, resp.) have been also reported in these men. 

As in part expected, in 2 of these cases, treatment with intramuscular testosterone did not give skeletal benefit to these patients, since aromatase deficiency does not lead to low testosterone levels [5, 47]. Conversely, estrogen treatment was associated with marked improvements in the skeletal phenotype. In fact, epiphyses closed quickly, longitudinal growth ceased, and BMD increased consistently at all assessed skeletal sites [41, 42, 47, 51, 52]. As counterpart, estrogen replacement in the male case of loss of function mutation at the *ESR1* gene did not improve bone outcomes [5, 50]. Of interest, consistent with the threshold estradiol hypothesis for skeletal health, a clear dose-dependent effect of estrogen replacement therapy on bone mass was evidenced, since a very low dose of estradiol (below 25 mcg twice weekly) was not sufficient for maintaining a normal BMD in one of these aromatase-deficient men [52]. 

In a more recent study, the skeletal phenotype of a 16-year-old boy with aromatase deficiency was investigated by both DXA and peripheral quantitative computed tomography (pQCT) of the radius [46]. The use of the later technique allowed to assess additional characteristics of bone strength, such as cross-sectional area (CSA), cortical thickness, trabecular volumetric BMD, and cortical volumetric BMD. Consistent with the previous observations, estrogen replacement in this boy was associated with the normalization of sex hormone concentrations, reduced bone turnover rate, and increases in lumbar spine (+23%) and femoral neck (+14%) areal BMD. However, the gain in volumetric BMD (either estimated by the calculation of the bone mineral apparent density from DXA or assessed directly by pQCT) was limited at the lumbar spine and even absent at the femoral neck and the radius. Conversely longitudinal bone growth, cross-sectional area, and cortical thickness (as measured by pQCT) increased significantly by 8.5%, 46%, and 12%, respectively. Thus, it was clear that the observed increase in areal BMD was mainly driven by an increase in bone size, rather than bone density, particularly at peripheral sites. Interestingly, these changes are similar to those associated with normal pubertal growth and support the notion that in growing bones, except for the spine true density does not increase [53, 54]. On the contrary, periosteal diameter continues to expand and cortical thickness increases during normal male puberty because of reduced endocortical expansion and accelerated periosteal apposition [55]. These effects lead to increased bone size, and have classically been attributed to androgens, accounting for the greater areal density that is typical of the male skeleton. In fact, when females enter puberty, periosteal apposition is inhibited, an action classically believed to be an estrogen effect. However, according to the effects of estrogen administration on cross-sectional area and cortical thickness in this young boy with aromatase deficiency [46], some actions on bone size, previously attributed to androgens, must at least in part be an estrogen effect. Thus, it is likely that a biphasic, dose-dependent effect of estrogen at the periosteum could exist. At low levels (as observed in males and in early pubertal females) estrogen may stimulate periosteal apposition and increase bone size, whereas at higher concentrations (as observed in late pubertal and adult females) estrogen may inhibit cross-sectional bone growth. 

Recently, the concomitant presence of mild hypogonadism in a man with aromatase deficiency has offered a useful model to study the effects of testosterone and estradiol replacement, separately or in combination [56]. As expected, in this man, estradiol treatment alone increased BMD with a greater gain than the one obtained with testosterone alone. However, the combination of testosterone (6 mg/day) and estrogen (25 mcg twice weekly) replacement led to a further increase in cortical thickness at the radius and the tibia as measured by pQCT, further supporting the concomitant importance of both sex steroids for periosteal apposition. In this case, an increase in volumetric BMD at the tibia and the radius as well as an increase of areal BMD of the lumbar spine and the femoral neck was also described after 2 years of combined therapy.

The skeletal consequences of aromatase deficiency have been illustrated further by studies of the aromatase knock-out mouse (ArKO) models [57–59]. Overall, these experimental observations generally provided the confirmation of the human gene disorders suggesting that estrogen may be more protective in the growing skeleton than androgens in man, but left open the issues of what, if any, are the roles of estrogen in regulating bone remodeling and bone loss in adult males.

### 3.2. Inhibition of Aromatase Activity: Clinical Studies in Adult Men

A first indirect indication about the importance of aromatase and the relative roles of estrogen versus testosterone in the adult male skeleton came from cross-sectional and longitudinal observations in middle-aged and elderly men. In fact, in most of these studies BMD and bone loss were more directly related to declining estrogen levels than declining androgen levels, particularly, when circulating bioavailable fractions of these steroids were considered [8–10, 60–66]. Moreover, in one of these studies, the ratio between estradiol and testosterone presumed to be an indirect index of aromatase activity, increased significantly with age, and was higher in normal than in osteoporotic subjects or in men with fragility fractures [10]. Consistent with these findings, other clinical evidences underlined the importance of estrogen on the adult male skeleton. Thus, in a preliminary observation, Taxel and Raisz described significant reductions in bone resorption markers in 9 elderly men treated short term with either 0.5 mg or 2.0 mg daily of micronized 17*β*-estradiol [67]. In a different study, Anderson et al. treated 21 eugonadal osteoporotic men with intramuscular testosterone and found a significant increase in lumbar spine BMD, which was correlated with changes in serum estradiol, but not circulating testosterone [68].

In order to definitively dissect out estrogen versus testosterone effects on the adult male skeleton, more dynamic short-term interventional observations have been performed, where aromatase activity was suppressed through the use of aromatase inhibitors. In a first study, Falahati-Nini et al. [69] examined the differential effects of estrogen versus testosterone replacement in a group of 59 elderly men (mean age 68 yr) following the induction of hypogonadism (by the use of a GnRH agonist, Lupron) and aromatase inhibition (with letrozole 2.5 mg daily). Of interest, the increase in bone resorption markers (deoxypyridinoline and N-telopeptide of type I collagen) associated with the use of the GnRH agonist was almost completely prevented by estradiol but not by testosterone therapy alone, indicating that the increase in bone resorption was due primarily to estrogen loss, not to testosterone loss. In the case of bone formation markers, there was evidence for stimulatory effects of both estrogen and testosterone on serum osteocalcin but not the amino-terminal propeptide of type I procollagen that only increased with estrogen replacement. Since osteocalcin is produced primarily by mature osteoblastic cells and osteocytes [70], these findings are consistent with an important role for both estrogen and testosterone in maintaining the functional integrity of these cells. Type I collagen, by contrast, is produced by cells of the entire osteoblastic lineage [71], and these data would suggest that it is primarily estrogen that regulates osteoblast differentiation. These results were in part replaced in a similar study performed in younger individuals, [72] following the induction of the hypogonadal state by the GnRH agonist, goserelin acetate. In this model, evidence was provided for independent effects of testosterone and estrogen on bone resorption. A subsequent study by Taxel et al. [73] with a longer observation period (9 weeks) also gave similar results, further indicating that treatment of elderly men with an aromatase inhibitor (in this specific-case anastrozole 2 mg/day) produces significant increases in bone resorption and decreases in bone formation. These effects on bone turnover may be less evident with lower doses (i.e., anastrozole 1 mg/day or less), or in case of borderline hypogonadism (testosterone levels less than 350 ng/dl), at least over a short-term period of treatment [74]. To this regard, a more recent study assessed the 12 months effects of a low dose of anastrozole (1.0 mg/day) on BMD and bone turnover markers in 69 men (aged 60 yr or older) with low or low-normal testosterone levels [75, 76]. Interestingly, with this longer observation period, despite an increase in testosterone levels at all time points, a statistically significant decrease in posterior-anterior spine BMD versus placebo was described, likely due to the parallel mild decrease in serum estradiol. Qualitatively similar changes, although nonsignificant, were observed at the other bone sites. Conversely, bone turnover markers were not significantly affected by aromatase inhibition, in this study.

## 4. Variation in Aromatase Activity and Bone Metabolism in Men

All the above clinical and experimental models (in which aromatase activity is absent or inhibited) have clearly shown that aromatase and estrogen production are important factors in male skeletal health. These models, however, were mainly based on conditions of complete estrogen deficiency, while they did not address the potential skeletal implications of interindividual variation in aromatase efficiency [5, 14]. Such differences in aromatase activity, and, hence, estrogen levels, might become particularly important in elderly males in whom age-related declines in testicular and adrenal androgen precursors are common. 

### 4.1. Inherited Variation in Aromatase Activity

Several polymorphic regions have been detected in the human *CYP19A1* gene that could be responsible for qualitative and/or quantitative differences in gene expression and aromatase activity (Figure 2). The most widely studied include a silent polymorphism (G/A at Val80) in exon 3, a tetranucleotide (TTTA)n tandem repeat polymorphism in intron 4, a Arg264Cys (C/T) substitution in exon 7, and a single nucleotide change (C/T) in exon 10. These polymorphisms were investigated in postmenopausal women or elderly men. In particular, several studies evidenced an association between the number of TTTA repeats and estrogen levels, breast cancer, or osteoporotic risk [77–83]. More recently other polymorphic variants within the promoter region of the *CYP19A1* gene have been widely investigated and associated with BMD in both genders as well as with susceptibility to breast or uterine cancer in females [84–86]. Most of these evidences were confirmed in subsequent meta-analyses and large scale studies [87–91]. Thus, it is possible that the presence of particular *CYP19A1* variants could be responsible of higher aromatase activity and increased estrogen production. If so, these polymorphisms should be protective for bone loss in elderly men or postmenopausal women while potentially also increasing the risk of estrogen-related cancer.

**Figure 3:**
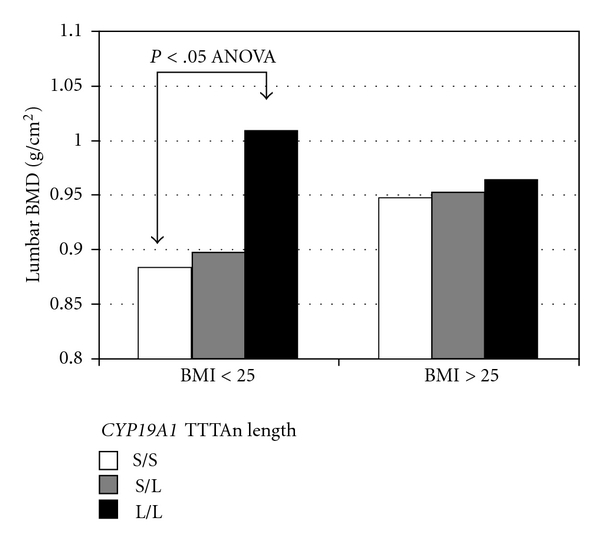
Lumbar BMD values according to *CYP19A1* (TTTA)n repeat genotype in subjects divided by BMI in normal (BMI ≤ 25) and overweight or obese groups (BMI > 25), respectively. Subjects were grouped according to short (S, TTTA ≤ 9) and long (L, TTTA > 9) repeats number (adapted from [81]).

Of interest, the skeletal consequences of genetic variation in* CYP19A1* appear to be modulated by fat mass, particularly in men. In fact, BMD differences associated with *CYP19A1* genotypes were greater in male subjects with a normal BMI, while the association progressively decreased when overweight and obese men were analyzed [81] (Figure 3). This point suggests that fat mass may be a mitigating factor in the expression of *CYP19A1* genotypes on bone. It is possible that with more adipose tissue, the associated increase in adipose aromatase activity dominates any effect of the polymorphisms on intrinsic aromatase activity. 

Given the importance of estrogen in bone growth, it is likely that genetic variation in *CYP19A1 *may be also relevant for young individuals, before the attainment of peak bone mass. Despite an initial study in 140 middle-aged Finnish men evidenced an association between the number of TTTA repeat sequences and height or BMI but not with BMD [92], a larger analysis in younger individuals confirmed that *CYP19A1* polymorphisms significantly affect the attainment of peak bone mass [93]. Thus, in a well-characterized cohort of 1068 men at the age of peak bone mass (18.9 ± 0.6 years), both the TTTA repeat variation and a silent G/A polymorphism at Val80 of the *CYP19A1* gene were predictors of areal BMD, lumbar spine, total body, and cortical bone size (cortical cross-sectional area and thickness) at 2 peripheral sites (radius and tibia).

To date, the molecular mechanisms through which the different *CYP19A1* variants affect aromatase activity, and bone metabolism remain in great part unknown. In a preliminary analysis, higher in vitro aromatase efficiency and greater estrogen production were observed in fibroblasts from men with a high TTTA repeat genotype than in men bearing a low TTTA repeat genotype [81]. Even though, it is unlikely that this polymorphism might have a direct effect on aromatase expression and activity (due to its intronic location within the *CYP19A1* gene), a different study described a strong degree of linkage disequilibrium between the TTTA repeat variation and the C-T substitution in exon 10, just 19 base-pairs downstream of the termination site of translation [78]. Interestingly, in that study, the T allele was associated with a higher number of TTTA repeats and showed a high-activity phenotype, with increased aromatase activity, increased aromatase mRNA levels, and with a switch in promoter usage from adipose tissue promoter to the more active ovary promoter. More recent studies also evidenced a functional role of other polymorphisms located within the complex promoter region of the *CYP19A1* gene. In particular, a C/G polymorphisms in promoter I.2 (rs1062033) were associated with differences in gene transcription by interacting with the transcription factor CEBP*β* [94], which affects aromatase expression in different tissues [95, 96]. In fact, the expression of the reporter luciferase gene in osteoblast cell lines was significantly higher in constructs bearing the G allele (which was also associated with higher BMD in population studies) than in those with the C allele, and this difference was particularly evident after cotransfection with CEBP*β*. Consistent with these results, differential allelic expression was also evidenced in bone tissue samples, again indicating the G allele as the more overexpressed. Although these studies, in the aggregate, provide data to argue for the importance of *CYP19A1 *polymorphisms as determinants of estrogen production and bone strength, larger and more definitive studies are needed before any firm conclusion can be drawn.

Interestingly, epigenetic effects on aromatase transcription and activity have been also evidenced in recent studies. In fact, CpG methylation has been described as an important epigenetic mechanism for the regulation of *CYP19A1* expression. To this regard, an in vitro study performed in skin fibroblast indicated that differences in methylation patterns may be responsible for the observed interindividual variation in promoter-driven expression of aromatase [97]. In particular, in this study, unmethylated constructs showed consistently higher promoter activity than methylated constructs.

### 4.2. Acquired Variation in Aromatase Activity

Besides the above genetic and epigenetic considerations, several additional mechanisms have been proposed in which aromatase activity could be modulated under certain circumstances in different tissues. It is known, for example, that aromatase is a specific marker of the undifferentiated adipose mesenchymal cell phenotype while it is less expressed in mature adipocytes. Thus, factors that stimulate adipocyte differentiation, such as PPAR*γ* agonists could also lead to the downregulation of aromatase gene and a reduction in aromatase activity [98–100]. Of course, if there are more adipocytes, there could be more aromatase activity even with reduced production of estrogen per fat cell. Moreover, the skeletal effects of PPAR*γ* agonists are more complex and mainly involve direct negative effects on bone cells [101]. Other agents acting on PPAR*γ* and PPAR*α* pathways, such as the phthalates (ubiquitous environmental toxins found in plasticizers) have been associated with a decrease in aromatase mRNA and aromatase activity in ovarian granulosa cells [102]. Moreover, the activation of PPAR*α* pathway by fenofibrate in female mice significantly reduced aromatase mRNA and activity and was associated with a decrease in femoral BMD and uterine size [103]. Despite these different experimental evidences, the clinical relevance of these environmental agents on global aromatase activity and estrogen production in man remains unknown. 

Several other contaminants may affect aromatase activity and estrogen production. Glyphosate-based herbicides are toxic and endocrine disruptors in human cell lines that are widely used across the world. Their residues are frequent pollutants in the environment and are spread on most eaten transgenic plants, modified to tolerate high levels of these compounds in their cells. While up to 400 ppm of these residues are accepted in some feed, recent experimental studies demonstrated that aromatase transcription and activity were disrupted with subagricultural doses and with residues from 10 ppm [105]. In addition, a different study indicated that phytochemicals such as procyanidin B dimers contained in red wine and grape seeds inhibits aromatase activity *in vitro* and suppress aromatase-mediated breast tumor formation *in vivo* [106]. To this regard, it has been assumed that daily consumption of 125 mL of red wine would provide adequate amounts of procyanidin B dimers to suppress in situ aromatase in an average postmenopausal woman. By a different mechanism myosmine, a minor tobacco alkaloid widely occurring in food products of plant and animal origin inhibits the conversion of testosterone to estradiol by human aromatase with potential implications for sex hormone homoeostasis [107]. Another important and well-recognized modulator of aromatase efficiency in bone cells is vitamin D that has been shown to stimulate glucocorticoid-induced aromatase activity in cultured osteoblasts [35]. The magnitude of this effect varies largely among individuals, depending on the level of the vitamin D receptor [107]. Of interest, vitamin D receptor knock-out mice showed reduced aromatase activity with respect to wild-type animals [108]. 

**Figure 4:**
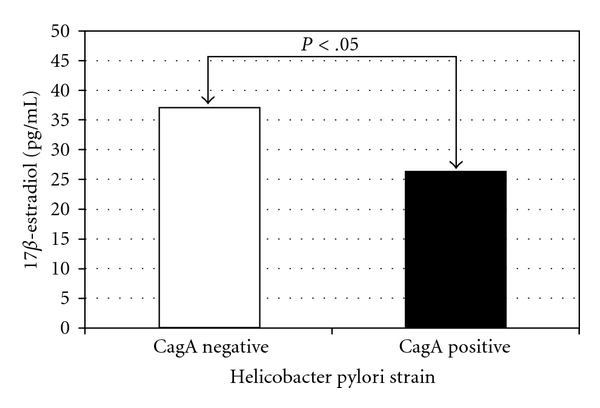
Variations in estrogens levels in elderly men affected by Helicobacter Pylori CagA positive or negative strains (adapted from [104]).

Importantly aromatase activity may be also affected by pathological conditions. In this respect, it is known that increased androgen aromatization can occur in case of hepatocellular carcinoma [109], adrenocortical tumors [110], and testicular tumors [111, 112]. In all these neoplastic conditions, inappropriate amounts of aromatase enzyme are expressed and estrogen levels are increased. Elevated plasma estradiol concentrations also have been described in men with liver cirrhosis together with decreased plasma testosterone [112, 113]. In these patients, the metabolic clearance rate of estrogens seems to be unaltered, suggesting that the observed hyperestrogenism could be caused solely by an increase in aromatization of androgen precursors. Conversely, other pathological conditions may negatively affect aromatase activity and estrogen levels in males. In a preliminary study on elderly men Figura et al. described significant differences in estradiol levels in relation to Helicobacter pylori infection, independently from circulating testosterone levels [105]. Serum concentrations of estradiol were significantly lower in infected CagA-positive patients than CagA-negative patients (Figure 4), and this variation was associated with differences in bone turnover. The mechanism underlying this association is unknown and deserves further investigations. Indeed, aromatase activity and production of estradiol were recently demonstrated in gastric parietal cells [114]. Finally, more recent observations suggested that diabetes may negatively affect expression levels of aromatase at least in the ovary and the testis [115, 116]. The effects of this disorder on major extragonadal sites of aromatase activity including bone remains to be determined. Of interest, experimental studies also evidenced that oral antidiabetic agents such as metformin can decrease aromatase expression in both granulose-luteal cells and breast adipose cells while, on the opposite, insulin has been associated with enhanced aromatase expression in different cell lines [117, 118]. Importantly, since a recent study evidenced that metformin-induced inhibition of aromatase expression occurs via the downregulation of promoter II, I.3, and 1.4 [118], its potential negative effects on skeletal estrogen production and bone health should be investigated. 

## 5. Conclusions

Extraglandular aromatization of circulating androgen precursors is the major source of estrogen in men. Several lines of clinical and experimental evidence now clearly indicate that aromatase activity and estrogen production are necessary in men (as well as in women) for longitudinal bone growth, the pubertal growth spurt, epiphyseal closure, normal bone remodeling, and the attainment of peak bone mass. Moreover, like in women, estrogen production from androgen precursors by peripheral aromatase activity (even within the bone) is also important for the maintenance of bone mass and the prevention of bone loss in aging men. Further studies are required to better understand how genetic, epigenetic, environmental, pathologic, and pharmacological influences might modulate aromatase activity, increasing or reducing estrogen production in ageing individuals, and thereby affecting skeletal health.
